# WheatCRISPR: a web-based guide RNA design tool for CRISPR/Cas9-mediated genome editing in wheat

**DOI:** 10.1186/s12870-019-2097-z

**Published:** 2019-11-06

**Authors:** Dustin Cram, Manoj Kulkarni, Miles Buchwaldt, Nandhakishore Rajagopalan, Pankaj Bhowmik, Kevin Rozwadowski, Isobel A. P. Parkin, Andrew G. Sharpe, Sateesh Kagale

**Affiliations:** 1 National Research Council Canada, 110 Gymnasium Place, Saskatoon, SK S7N 0W9 Canada; 2 Agriculture and Agri-Food Canada, 107 Science Place, Saskatoon, SK S7N 0X2 Canada; 3 Global Institute for Food Security, University of Saskatchewan, 110 Gymnasium Place, Saskatoon, SK S7N 4J8 Canada

**Keywords:** CRISPR, Cas9, gRNA design tool, Genome editing, Transcriptional regulation, Wheat

## Abstract

**Background:**

CRISPR/Cas9 gene editing has become a revolutionary technique for crop improvement as it can facilitate fast and efficient genetic changes without the retention of transgene components in the final plant line. Lack of robust bioinformatics tools to facilitate the design of highly specific functional guide RNAs (gRNAs) and prediction of off-target sites in wheat is currently an obstacle to effective application of CRISPR technology to wheat improvement.

**Description:**

We have developed a web-based bioinformatics tool to design specific gRNAs for genome editing and transcriptional regulation of gene expression in wheat. A collaborative study between the Broad Institute and Microsoft Research used large-scale empirical evidence to devise algorithms (Doech et al., 2016, Nature Biotechnology 34, 184–191) for predicting the on-target activity and off-target potential of CRISPR/*Sp*Cas9 (*Streptococcus pyogenes* Cas9). We applied these prediction models to determine on-target specificity and potential off-target activity for individual gRNAs targeting specific loci in the wheat genome. The genome-wide gRNA mappings and the corresponding Doench scores predictive of the on-target and off-target activities were used to create a gRNA database which was used as a data source for the web application termed WheatCRISPR.

**Conclusion:**

The WheatCRISPR tool allows researchers to browse all possible gRNAs targeting a gene or sequence of interest and select effective gRNAs based on their predicted high on-target and low off-target activity scores, as well as other characteristics such as position within the targeted gene. It is publicly available at https://crispr.bioinfo.nrc.ca/WheatCrispr/.

## Background

Genome editing technology based on a bacterial adaptive immune system, termed CRISPR (Clustered, Regularly Interspersed, Palindromic Repeats) / Cas9 (CRISPR-associated endonuclease 9 [1–4];) has sparked a new revolution in biological and agricultural research [5, 6]. CRISPR/Cas9 technology originating from *Streptococcus pyogenes* relies on two important components, a Cas9 endonuclease and a single guide RNA (sgRNA) formed by fusing two small RNA molecules, namely CRISPR RNA (crRNA) and an auxiliary trans-activating crRNA (tracrRNA) that together guide Cas9 nuclease to a specific DNA site [7, 8]. Each crRNA unit contains a 20-nt guide sequence complementary to a target site, designated as guide RNA (gRNA). Another critical feature of the Cas9 system is the Protospacer-Adjacent Motif (PAM) flanking the 3′-end of the DNA target site that dictates the target search mechanism of Cas9 [9]. The PAM comprises a triplet of base pairs with a canonical sequence 5′-NGG-3′ where “N” is any nucleotide [9]. Other non-canonical PAM triplets have also been described, including NAG, NCG and NGA that support less efficient CRISPR/Cas9 functions [10, 11], and thus may contribute to off-target activity.

Although CRISPR/Cas9 applications promise to accelerate the pace and course of crop improvement [5, 6], a number of hurdles exist that limit full exploitation of this innovative technology, especially in crops with large polyploid genomes. Wheat is an economically important cereal crop providing 20% of the calorie and protein intake for the global population. It harbours a complex allohexaploid genome of 16 Gb with approximately 85% repetitive elements and estimated 107,921 high confidence and a further 161,537 low confidence annotated genes [12]. Due to the presence of up to six homoeoalleles per gene and large gene families, off-target gRNA binding and cleavage is one of the most critical issues that affect implementation of CRISPR/Cas9 technology in wheat. The gRNA is an important component of the CRISPR/Cas9 system as it determines the efficacy and specificity of Cas9 nuclease. An effective gRNA should have high on-target activity and low off-target potential. Thus, rational design and optimization of functional gRNA sequences is essential to achieving maximal effectiveness and highest targeting specificity for intended genomic location(s).

Multiple bioinformatics tools have been developed to facilitate the design of gRNAs and prediction of off-target sites [13–24]; however, only two of these programs, including E-CRISP [17] and CRISPRdirect [19] support design of gRNAs for wheat. CRISPRdirect predicts specific gRNAs based on in silico prediction of specificity but the lack of implementation of evidence-based metrics to predict off-target sites is a notable caveat. E-CRISP identifies off-target sites by aligning gRNAs to the genome with Bowtie2. However, Bowtie2 does not guarantee that all possible hits will be found, especially when the number of mismatches is high [11]. This results in an underestimation of potential off-target sites. A collaborative effort between scientists at the Broad Institute and machine learning experts at Microsoft Research used large-scale empirical evidence based on cleavage potential of thousands of gRNAs targeting a panel of 15 genes to uncover position-specific sequence features that are predictive of gRNA efficacy and specificity, including the position and frequency of single and di- nucleotides, the GC content of the gRNA, the location of the gRNA within the protein coding region and melting temperatures of the first 5, middle 8 and last 5 base pairs of the gRNA [11, 25]. The findings from these large-scale empirical data were utilized to devise new rules for gRNA on-target activity [rule set (rs) 2] and cutting frequency determination (CFD) scores to predict gRNA off-target effects [11], that can be broadly applied. In this study, we applied these prediction models to determine on-target specificity and potential off-target activity of individual gRNAs targeting any locus in the wheat genome, and designed a web-based bioinformatics portal (WheatCRISPR) for design of highly specific gRNAs for CRISPR/Cas9-mediated genome editing and CRISPR-based transcriptional regulation of gene expression in Chinese Spring wheat.

### gRNA database construction and content

Based on the current annotation [12], the bread wheat genome has approximately 35 million canonical PAM sites in coding regions and over 6 billion potential off-target sites across the entire genome including intergenic regions and non-canonical PAM sites (Table 1). Although applying the Doench algorithms is conceptually simple, the huge number of PAM sites in the large wheat genome makes the task of predicting off-target activity computationally challenging. Running the prediction models on all possible pairs of on-target (canonical coding and promoter) and off-target (all) sites is a daunting computational endeavour.

**Table 1:** Survey of PAM sites in the IWGSC v1.0 Chinese Spring wheat reference genome sequence

Region	Canonical (NGG)	Non-canonical (NAG, NCG and NGA)
Coding	33,543,850	93,876,010
Promoter	46,236,876	133,448,536
UTR/Intron	32,345,815	101,719,313
Intergenic	1,520,739,628	4,374,331,934

**Fig. 1:**
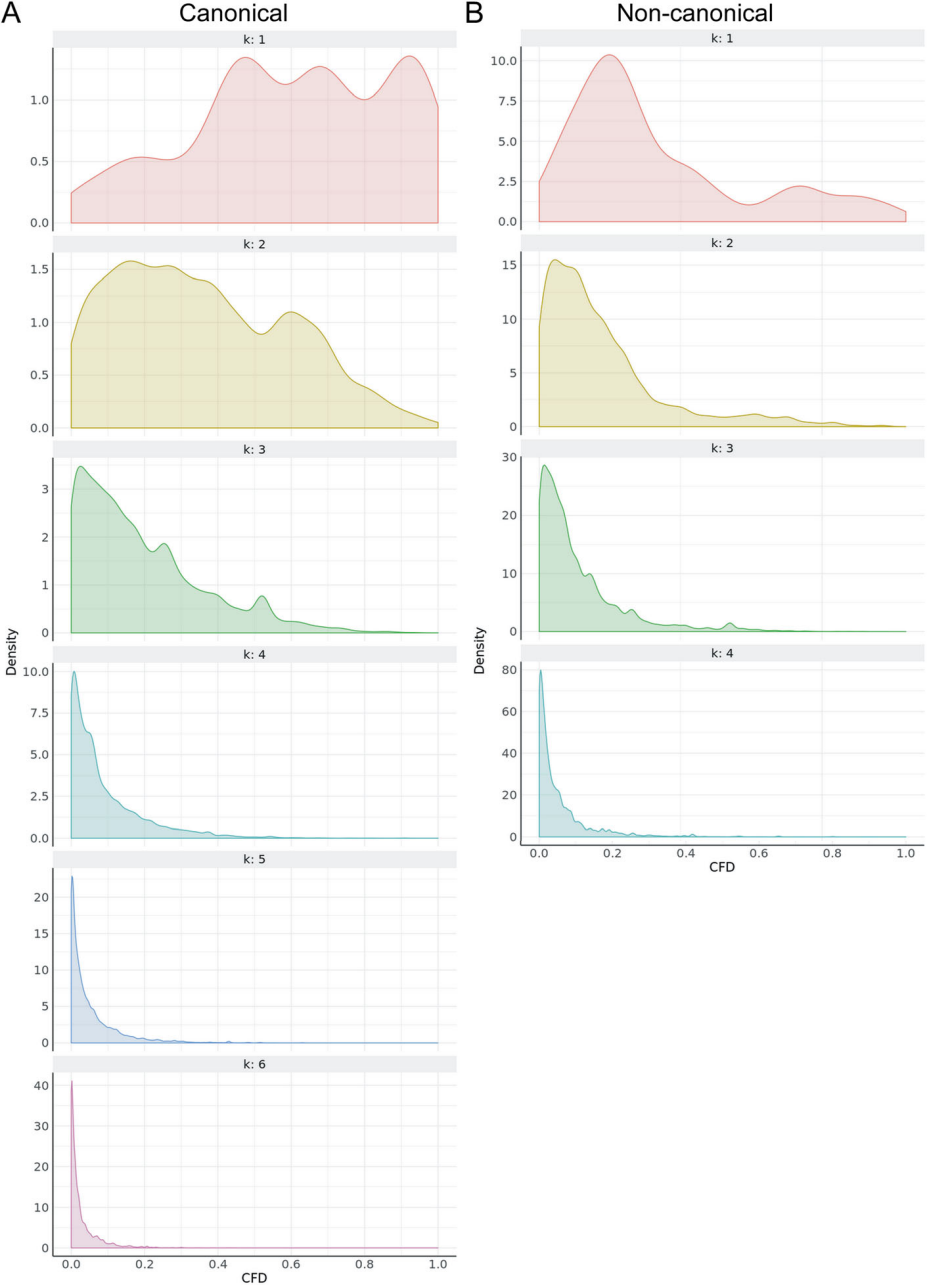
Assessment of off-target effects of gRNAs with a tiered *k* (mismatches) approach. CFD densities are plotted by *k* (mismatches) using a subset of approximately one million canonical (**a**) and non-canonical (**b**) on-target::off-target gRNA pairings

To reduce the number of potential off-target sites that must be considered to apply the Doench CFD algorithm, we limited our search to only those sites that have at most *k* mismatches to on-target sites. While searching for sites with *k* mismatches is much faster than applying the Doench algorithm to every possible site, it is still computationally intensive. To make this solution more tractable, we varied the maximum value of *k* in different regions of the genome such that the likelihood of detrimental off-target effects is minimized. For canonical NGG PAM sites in exons and promoter regions (2 kb upstream of the start codon), we searched up to *k* = 6. For other genic regions [i.e. introns and untranslated region (UTR)], we searched up to *k* = 4, and for intergenic regions we searched up to *k* = 3 **(**Table 2**)**. For non-canonical PAM sites (NAG, NCG, NGA) we searched at *k* = 4, *k* = 3, and *k* = 2 in these regions, respectively **(**Table 2**)**. In addition to these absolute *k* limits in each region, if at least 20 off-target matches were found up to a given *k* mismatches, we did not proceed to search for *k* + 1 mismatches. Testing a subset of 1 M on-target::off-target gRNA pairings suggested that the wheat genome has very few active off-target sites with *k* ≥ 3 **(**Fig. 1**)**, making the tiered *k* approach a far more efficient search mechanism for the large wheat genome.

**Table 2:** Tiered *k* (mismatches) levels applied for the search of off-targets in the wheat genome

PAM	Off-target region	*k* (mismatches)
Canonical (NGG)	Exons and promoters	6
	Introns and UTRs	4
	Intergenic	3
Non-canonical (NAG, NCG, NGA)	Exons and promoters	4
	Introns and UTRs	3
	Intergenic	2

**Fig. 2:**
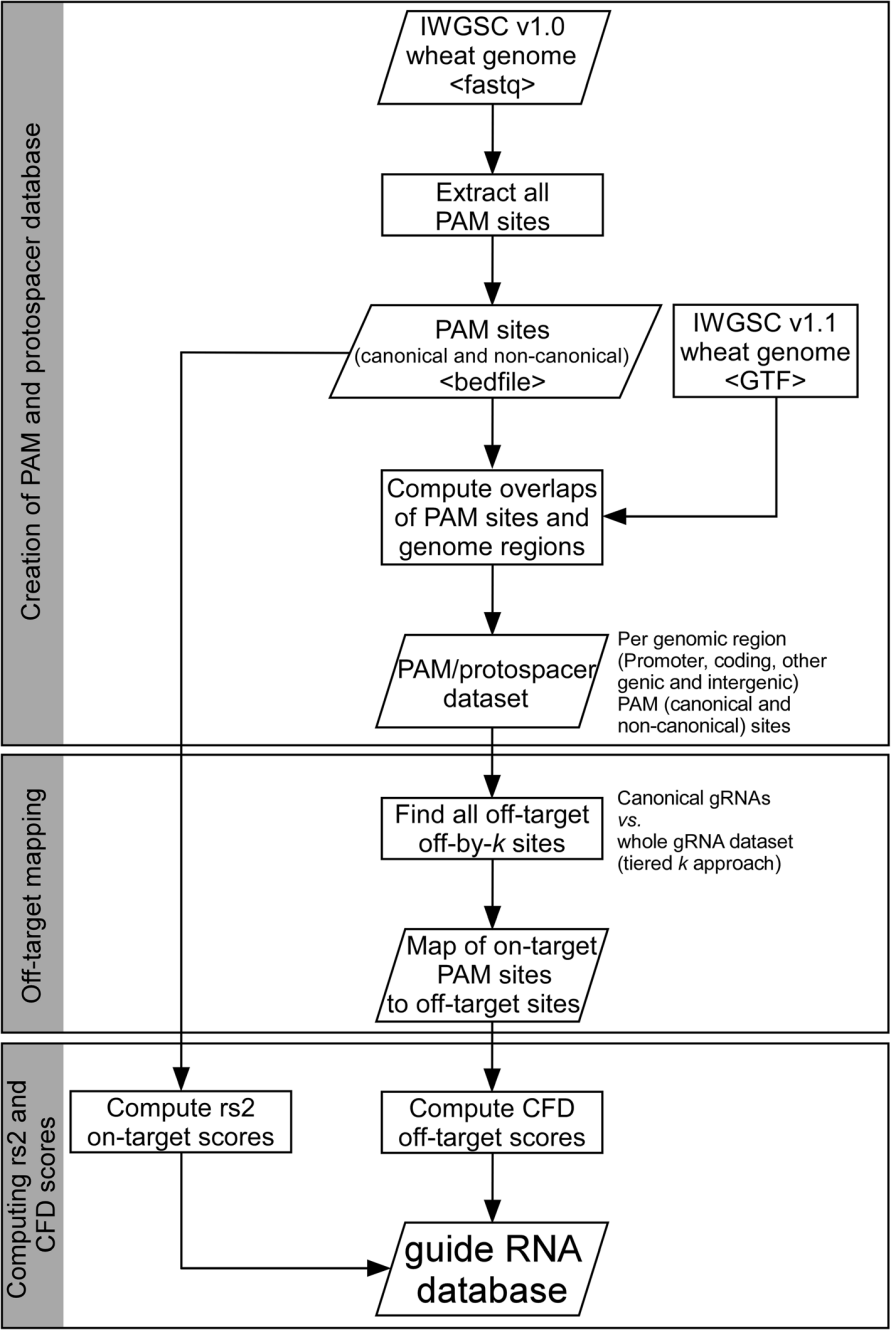
Flowchart of the steps involved in creating the gRNA database. The workflow comprises three major steps, including (1) genome-wide scanning and extraction of PAM sites and adjoining gRNA sequences, (2) mapping off-targets and (3) computing on-target activity (rs2) and cutting frequency determination (CFD) scores based on the Doench prediction models (Doench et al., 2016)

To implement this strategy, we extracted the PAM and gRNA sequences from all possible PAM sites in the IWGSC v1.0 wheat (Chinese Spring) genome [12], separating the output into canonical and non-canonical sites. These sites were further categorized by their genomic location: coding, promoter, other genic (introns and UTR), and intergenic. The canonical coding and promoter sets were designated as on-target datasets and the rs2 algorithm was applied to each on-target gRNA sequence. Each of the on-target gRNA sequences was then searched against all eight datasets for off-by *k* mismatches, setting the maximum *k* as described in Table 2. The Doench CFD algorithm was applied to the resulting set of on-target::off-target mappings. The steps involved in creating the gRNA database are illustrated in Fig. 2**.** The gRNA mappings, the Doench scores, and the positions of the sequences in the genome were used as the data source for the WheatCRISPR web application.

### Utility and discussion

**Fig. 3:**
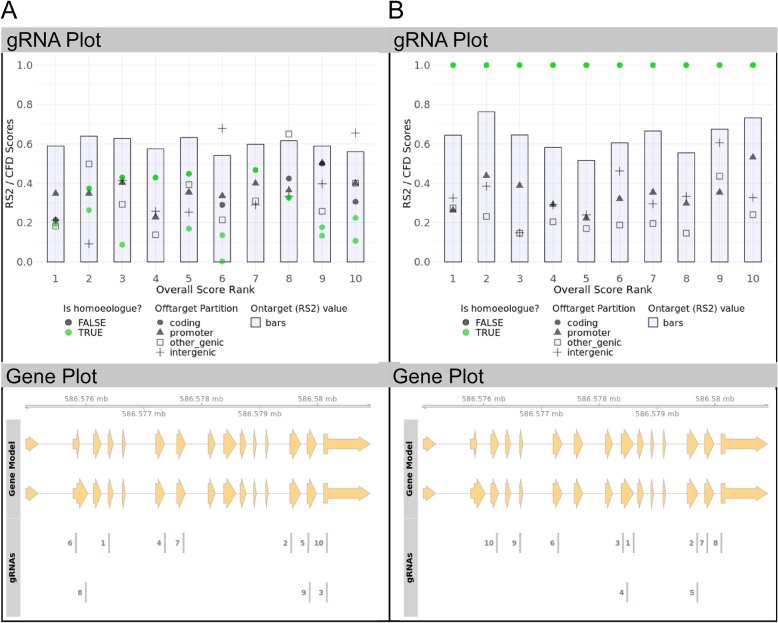
Outputs from the WheatCRISPR web application. Exemplary gRNA and gene plots for the phyotoene desaturase (PDS) produced using the WheatCRISPR web application are shown: **a** gRNAs specifically targeting TraesCS4B02G300100, and **b** gRNAs targeting all three homoeologues of the PDS gene (TraesCS4B02G300100, TraesCS4A02G004900, TraesCS4D02G299000). The gRNA plot displays a visualization of the rs2 and CFD scores and the score for any off-target hits to homoeologues. The blue-gray bars show the rs2 (on-target activity) score, the black points indicate the worst CFD (off-target activity) scores for each region (coding, promoter, other genic, intergenic), and the green points, if any, show the CFD scores for homoeologues. The gene plot displays the physical location of the gRNAs against the gene models. Each row in the gene model represents an isoform of the gene. The gray lines indicate introns and orange bars indicate exons. The thinner orange bars indicate UTRs

WheatCRISPR (https://crispr.bioinfo.nrc.ca/WheatCrispr/) provides a convenient interface to browse the gRNA database, and allows researchers to view a set of predicted gRNAs targeting a gene or sequence of interest, and select them based on their predicted on-target and off-target activity, and the position of the gRNA within the targeted gene. The application presents summary statistics in graphs and tables that expedites the quick finding of the most effective candidate gRNAs for the gene of interest (Fig. 3 and Additional file 1: Table S1). By default, detailed information is displayed for the ten highest scoring gRNAs to facilitate rapid identification of the most likely efficacious candidate gRNA sequences. An interactive interface allows the user to browse all other gRNAs if desired.

A key summary statistic for evaluating the off-target activity of a gRNA is the maximum CFD score for the gRNA, i.e. the single worst off-target hit. The gRNA plot (exemplified in Fig. 3a) and table (Additional file 1: Table S1) for a given gene presents the rs2 score and the maximum CFD score for each of the four genomic regions: coding, promoter, other genic, and intergenic. This facilitates selection of specific gRNAs by characterizing the potential severity of off-target effects based on the likelihood of unintended activity resulting in functional change to a coding region.

WheatCRISPR assists the user to find a trade-off between high on-target activity and low off-target activity by calculating an overall score for each gRNA that rewards high rs2 scores and penalizes high CFD scores (Additional file 1: Table S1). The overall score is a weighted average of the rs2 and maximum CFD scores. An optional variation of this score can be toggled on or off if the user wishes to target all homoeologous copies of the gene. In this variation, high CFD scores in homoeologues are rewarded while the maximum CFD in non-homoeologues remains penalized (Fig. 3b). Homoeologs were identified by the annotation available at ensemblgenomes.org. An overall score is used to rank all gRNAs for a gene so that the user can quickly identify the most likely candidate gRNAs. The overall scoring function is not based on any empirical evidence, so it is simply an intuitive estimate designed to help accelerate the process of finding effective gRNAs. Users are strongly encouraged to consider the individual rs2 and CFD scores, and other factors such as the location of gRNA within the protein coding region of the gene, before selecting a gRNA. The exact function used when targeting a specific gene (the default mode) is:

0.5(rs2) + 1 − (0.5(0.7(max(cfd_coding,cfd_promoter)) + 0.2(max(cfd_other_genic)) + 0.1(max(cfd_intergenic)))0.5(rs2) + 1-(0.5(0.7(max(cfd_coding,cfd_promoter)) + 0.2(max(cfd_other_genic)) + 0.1(max(cfd_intergenic)))

and when targeting homoeologs is enabled:

0.33(rs2) + (1 − (0.33(0.7(max(cfd_coding,cfd_promoter)) + 0.2(max(cfd_other_genic)) + 0.1(max(cfd_intergenic))))) + 0.34(mean(cfd_hmlgs))

Besides the predicted on-target and off-target activity metrics, the location of the gRNAs within a gene can also be important. It is often desirable to select gRNAs from exons that occur in all splice isoforms of a gene to ensure that all alternative transcripts are targeted. To identify the location of gRNAs within a gene, WheatCRISPR presents a genome browser-style Gene Plot with tracks for the gene models and the selected gRNA **(**Fig. 3**)**.

The precomputed on-target to off-target mappings improve performance but limit the target sites to annotated genes. To search for targets outside annotated genes, WheatCRISPR also allows the user to paste in an arbitrary sequence of interest. In such cases, gRNAs are extracted, and off-target sequences and scores are computed on the fly. In this mode, functionality is limited for performance reasons. The maximum number of mismatches is limited as described in Table 2, and targeting a set of homoeologs is not possible.

**Fig. 4:**
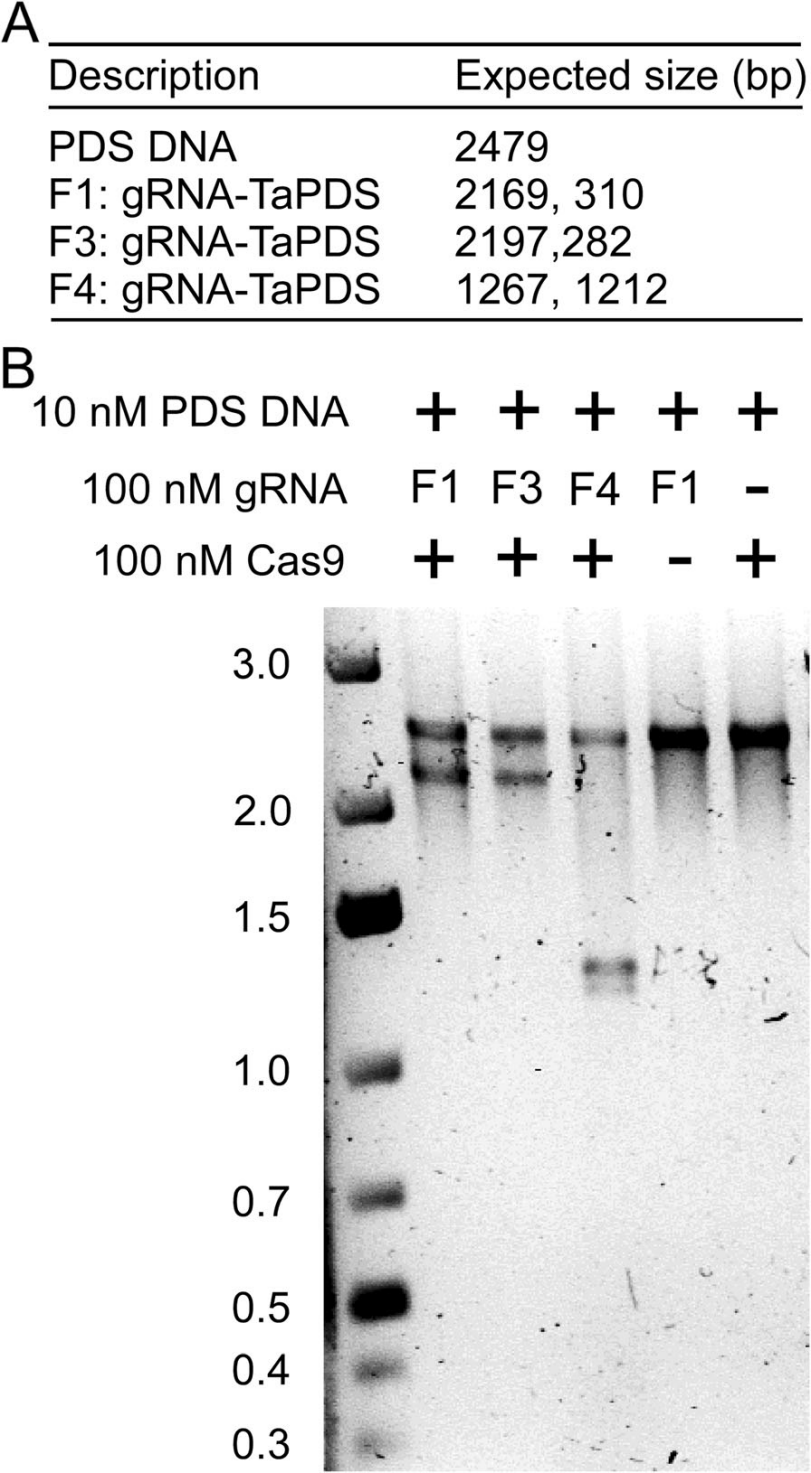
Functional validation of TaPDS gRNAs by in vitro nuclease assay. A 2.479 kb fragment of wheat TaPDS (TraesCS4B02G300100) gene was amplified using wheat (Chinese Spring) genomic DNA and the primers TaPDS_F3 (5′- cgcagaggtgtttcacaagt - 3′) and TaPDS_R4 (5′ - gagccatgcttctcctacac - 3′). **a** The expected band sizes of cleaved products using different guide RNAs against the 2.479 Kb input DNA. **b** In vitro nuclease assay of the input DNA using Cas9 and different guide RNAs. The Cas9 endonuclease (100 nM), guide RNAs (100 nM) and PCR amplified TaPDS target DNA fragment (10 nM) were mixed together at a molar ratio of 10:10:1 in a total assay reaction volume of 30 μl. The reactions were incubated at 37 °C for 30 min. The assay was stopped by the addition of Proteinase K and products were analyzed using an agarose gel electrophoresis

To validate the accuracy of prediction of gRNA efficacy by WheatCRISPR, we compared the overall ranking of a subset of gRNAs and their targeting efficiency reported in the literature (Additional file 2: Table S2) [26–29]. The wheat gRNAs reported to be successful in targeting Q (the spelt factor gene), TaGW2 (wheat grain width and weight 2), TaLpx1 (wheat lipoxygense 1), TaPDS1 (wheat phytoene desaturase 1) and INOX (inositol oxygenase) were predicted to have high on-target specificity and low off-target activity (ie., ranked higher based on WheatCRISPR overall score). On the contrary, gRNAs that either failed or had low success in editing TaGW2, TaDEP1 (wheat dense and erect panicle 1) and TaPIN1 (wheat PIN-FORMED 1) ranked lower (Additional file 2: Table S2). We have also confirmed the functionality of a number of gRNAs targeting wheat genes, such as TaPDS **(**Fig. 4**)** and puroindoline A [30], using an in vitro nuclease assay. These examples validate the prediction accuracy of WheatCRISPR and demonstrate its utility in genome editing applications in wheat.

## Conclusion

As an elegant alternative to reliance on natural or induced mutagenesis, CRISPR/Cas9-based gene editing technology has the potential to change the pace and course of crop breeding. To facilitate the application of this innovative technology in wheat, we have developed a robust web-based bioinformatics tool (WheatCRISPR) to enable selection of specific gRNAs for user-specified target gene or sequence and prediction of potential off-target sites. The current implementation of WheatCRISPR supports the selection of gRNAs to guide *S. pyogenes* Cas9 to genomic locations in the wheat genome. Identification of guide sequences with different PAMs reported for Cas9 variants, such as StCas9 (*Streptococcus thermophilus* Cas9), NmCas9 (*Neisseria meningitides* Cas9), SaCas9 (*Staphylococcus aureus* Cas9) and FnCpf1 (*Francisella novicida* RNA-guided endonuclease) would be highly desirable. However, the reliance of Doench algorithms on empirical data (gRNA efficacy and specificity) specific to PAM sites of SpCas9 limits extension of WheatCRISPR to PAM sites of other Cas9 variants. Additionally, in wheat there will be a few genes for which finding unique gRNAs would be difficult due to polyploidy, high content of repetitive DNA content and genes typically existing as members of multi-gene families with high levels of sequence identity. In such cases, the users may have to consider other strategies (for example, dual gRNAs) to improve targeting specificity.

## Availability and requirements

The WheatCRISPR web application is publicly available at https://crispr.bioinfo.nrc.ca/WheatCrispr/.

## Supplementary information

**Additional file 1: Table S1.** The gRNA table for a phytoene desaturase (TraesCS4B02G300100) gene.

**Additional file 2: Table S2.** Experimental corroboration and validation of gRNA efficiency predictions made by WheatCRISPR.

## Data Availability

All data generated or analysed in the study are included in this published article [and its supplementary information files].

## Abbreviations

Cas9: CRISPR-associated endonuclease
CFD: Cutting frequency determination
CRISPR: Clustered, regularly interspersed, palindromic repeats
crRNA: CRISPR RNA
gRNA: Guide RNA
PAM: Protospacer-Adjacent Motif
rs2: Rule set 2
tracrRNA: trans-activating crRNA
UTR: Untranslated region
